# Functional and structural changes in the neuroretina are accompanied by mitochondrial dysfunction in a type 2 diabetic mouse model

**DOI:** 10.1186/s40662-023-00353-2

**Published:** 2023-09-01

**Authors:** Christie Hang-I Lam, Bing Zou, Henry Ho-Lung Chan, Dennis Yan-Yin Tse

**Affiliations:** 1 School of Optometry, The Hong Kong Polytechnic University, Hong Kong, SAR China; 2 Centre for Eye and Vision Research Limited (CEVR), Hong Kong, SAR China; 3 Research Centre for SHARP Vision (RCSV), The Hong Kong Polytechnic University, Hong Kong, SAR China

**Keywords:** Diabetic retinopathy, Neurodegeneration, Mitochondrial dysfunction, Animal model

## Abstract

**Background:**

Diabetic retinopathy (DR), one of the leading causes of blindness and vision impairment, is suggested to exhibit functional and structural changes in retinal neurons as the earliest manifestation, which could be used to predict the progression of related angiopathy. While neural function and survival rely on proper mitochondrial function, and a growing body of literature has supported the role of mitochondrial dysfunction in the development of DR, how diabetes affects mitochondrial function in retinal tissue remains elusive. This study primarily aimed to investigate mitochondrial functional changes in a diabetic rodent model. We also characterized the early DR phenotype, in particular, neurodegeneration.

**Methods:**

C57BLKsJ-db/db (db/db) mice (a type 2 diabetic mouse model) were used with their normoglycemic heterozygous littermates (db/+) serving as controls. Longitudinal changes in retinal function and morphology were assessed with electroretinography (ERG) and optical coherence tomography (OCT), respectively, at 9, 13, 17, and 25 weeks of age. At 25 weeks, the retinas were harvested for immunohistochemistry and ex vivo mitochondrial bioenergetics.

**Results:**

Decreased ERG responses were observed in db/db mice as early as 13 weeks of age. OCT revealed that db/db mice had significantly thinner retinas than the controls. Immunohistochemistry showed that the retinas of the db/db mice at 25 weeks were thinner at the outer and inner nuclear layers, with lower photoreceptor and cone cell densities compared with the db/+ mice. The number of rod-bipolar cell dendritic boutons and axon terminals was significantly reduced in db/db mice relative to the db/+ mice, suggesting that diabetes may lead to compromised synaptic connectivity. More importantly, the retinas of db/db mice had weaker mitochondrial functions than the controls.

**Conclusions:**

Our longitudinal data suggest that diabetes-induced functional deterioration and morphological changes were accompanied by reduced mitochondrial function in the retina of db/db mice. These findings suggest that mitochondrial dysfunction may be a contributing factor triggering the development of DR. While the underlying mechanistic cause remains elusive, the db/db mice could be a useful animal model for testing potential treatment regimens targeting neurodegeneration in DR.

**Supplementary Information:**

The online version contains supplementary material available at 10.1186/s40662-023-00353-2.

## Background

Diabetes mellitus (referred to as diabetes below) is a chronic progressive disease characterized by uncontrolled hyperglycemia and a series of metabolic disorders. Obesity and sedentary lifestyle, related to economic development and urbanization, have been found to be major risk factors of this disease [1]. Despite the high prevalence of diabetes in developed countries, a recent report has indicated that the prevalence of diabetes is currently increasing most markedly in low- and middle-income countries [2]. According to the International Diabetes Federation, the number of diabetic patients will increase from 451 million in 2017 to 693 million by 2045 [3]. The rapidly increasing prevalence of diabetes and its related complications do not only affect the morbidity and mortality of patients, but also bring huge medical and economic burdens to society worldwide.

Diabetes adversely affects the physiology of many organs of the body, leading to a number of complications, among which diabetic retinopathy (DR) is one of the most common in diabetic patients [4–6]. Despite the recent advancements in treatment (in particular anti-vascular endothelial growth factor agents) and efforts in early detection of the disease, DR was still ranked in the top five leading causes of blindness and vision impairment, together with uncorrected refractive error, cataracts, glaucoma, and age-related macular degeneration, in adults aged 50 years and older in 2020. Notably, DR is the only cause that shows an increase in age-standardized prevalence [7].

Current diagnosis, severity grading, and treatment for DR ultimately focus on the related vascular lesions. However, diabetes affects the entire neurovascular unit of the retina, and a growing body of evidence suggests that diabetes-induced retinal neurodegeneration may occur prior to overt vascular abnormalities and could contribute to the development of vasculopathy [8–12]. Importantly, diabetes-induced damage to retinal neural cells and visual function, which may not be revealed by conventional fundus assessments, cannot be readily reversed. Thus, to preserve vision, it is imperative to develop new treatment regimens to arrest the development of DR at an early stage to prevent it from progressing to its proliferative stage.

Mitochondrial dysfunction has emerged as a potential mechanism contributing to the pathogenesis of DR. It has been implicated in cell death processes that occur in a range of retinal cells, including photoreceptor cells, Müller cells, endothelial cells, and pericytes, subjected to simulated hyperglycemia [13–16]. Changes in mitochondrial membrane structure in the microvasculature and expression of mitochondria-related genes have been reported in the retinas of diabetic rats 6–12 months after onset of diabetes and in patients with long-term diabetes [17]. However, how diabetes affects mitochondrial function of the retina at tissue levels is not well understood.

Neural function and survival rely on proper mitochondrial function for cellular adenosine triphosphate (ATP) production and calcium storage and buffering [18]. Disruption of mitochondrial homeostasis and subsequent mitochondrial dysfunction are closely related to the pathophysiology of neurodegenerative diseases, including but not limited to Parkinson's Disease and Huntington's Disease [19]. This study primarily aimed to investigate the effect of diabetes on retinal mitochondrial bioenergetics during the early development of DR. In addition, we longitudinally characterized the early DR phenotype in respect to neurodegeneration in C57BLKsJ-db/db (db/db) mice. Unlike other commonly used diabetic rodent models, including STZ-induced rodents and Ins2^Akita^ mice, the db/db mouse is a model of type 2 diabetes, the major subtype of diabetes worldwide.

## Methods

### Animal handling

Male db/db mice and their normoglycemic heterozygous littermates (db/+), obtained from The Chinese University of Hong Kong, were kept in the Central Animal Facility of The Hong Kong Polytechnic University, housed in groups of two in plastic cages with free access to standard rodent diet and water under a 12 h/12 h light–dark cycle. The experimental protocol was approved by the Animal Subjects Ethics Sub-committee (ASESC) of The Hong Kong Polytechnic University (Approval No. 19-20/85-SO-R).

### Evaluation of diabetes-related indices

The db/db mice are a model of type 2 diabetes with a spontaneous mutation of the leptin receptor gene, which leads to polyphagia, obesity, insulin resistance, and sequential diabetes and have been reported to commence development of diabetes at 4–8 weeks of age [20, 21]. At 9 weeks of age, blood glucose levels of the mice in the two groups were measured with a digital blood glucometer (Accu-Chek® Performa, Roche Diagnostics, Basel, Switzerland) after overnight fasting and db/db mice with a fasting blood glucose level ≥ 13.9 mmol/L were included in the study. Subsequently, the fasting blood glucose levels were measured every four weeks until 25 weeks of age. Hemoglobin A_1c_ (HbA_1c_) levels, which reflect diabetic status over three to four months, were measured at 9 and 25 weeks of age using DCA® Vantage Systems (Siemens Healthcare GmbH, Erlangen, Germany) with blood samples collected from the tail veins. All assessments were conducted between 9 a.m. and 10 a.m. Bodyweight was monitored weekly. Food and water consumption was measured thrice weekly.

### Electroretinography (ERG)

The in-vivo retinal functions were assessed with scotopic full-field ERGs (ffERG) as described previously [22] at 9, 13, 17, and 25 weeks. The mice were dark-adapted overnight (at least 16 h) in the experimental room prior to the ERG with all subsequent handling and preparations performed under dim red light. The animals were anaesthetized with a weight-based intraperitoneal injection of a solution containing Ketamine 100 mg/ml (Alfasan International BV, Woerden, Holland) and Xylazine 20 mg/ml (Alfasan International BV). The corneas of both eyes were anesthetized with Proxymetacaine 0.5% (Provain-POS®, Ursapharm, Saarbrücken, Germany), and the pupils dilated using a drop of the solution mixed with Tropicamide 0.5% and Phenylephrine 0.5% (Mydrin®-P, Santen, Osaka, Japan). The animals were then placed on a warm table maintained at 37 °C. A gold ring electrode was placed in contact with the cornea as the active electrode. Two platinum needle electrodes were inserted subcutaneously at the base of the tail and the forehead as the ground and reference electrodes, respectively. The impedances of the active and reference electrodes were less than 10 kΩ. A drop of 3% Carbomer 974P gel (Lacryvisc®, Alcon, Geneva, Switzerland) was applied to the cornea to prevent corneal dehydration. After positioning the electrodes, the animals were allowed to remain in complete darkness for 5 min before the start of the experiment.

Visual stimuli with white light-emitting diodes were delivered by a Ganzfeld bowl (Q450, Roland Consult, Brandenburg, Germany). Stimulation and data recording were performed using the RETI-Port system® (Roland Consult) according to a customized protocol, with stimulus intensities varying from − 4.32 log cd·s/m^2^ to + 1.3 log cd·s/m^2^. The signals were amplified and band-pass filtered from 1 to 30 Hz and 1 to 1000 Hz for scotopic threshold response (STR), which is suggested to originate from the proximal retina [23], and scotopic a- and b-waves, respectively. The b-wave of the scotopic ERG reflects the function of photoreceptors and the post-receptor pathway [24]. At the lowest intensity, 40 sweeps of response with a stimulus frequency of 0.5 Hz were averaged. The number of sweeps and stimulus frequency was reduced at higher flash intensity levels. The stimulus intensities were converted to the unit photoisomerizations/rod (R*/rod), where 1 scot cd/m^2^ = 516 R*/rod/s, after being calibrated by a photometer (ILT1700, International Light Technologies, Inc, Peabody, MA, USA), to determine the light zone to which the stimuli belonged, according to Abd-El-Barr et al. [25]. The responses triggered by the stimuli that were within the operation range of the rod cells (less than − 0.3 log cd·s/m^2^) were fitted with a sigmoidal curve, using the Naka-Ruston equation to determine the maximum b-wave response (Bmax).

Oscillatory potentials (OPs) were isolated by digital filtering of the raw signal recorded at + 0.3 log cd·s/m^2^ using a fast Fourier transform (FFT) and subsequent inverse FFT with an algorithm computed in the free software environment *R* (R Development Core Team, v.4.0.4). The raw data was first converted from the time domain to frequency domain. Spectral components beyond the cut-off frequency (65 to 300 Hz) [26] were then eliminated. The inverse FFT was performed to reconstruct the OP waveform in the time domain. Implicit times of OP were measured from the stimulus onset to the peak of the OP. Individual OP amplitude was measured from the peak to the adjacent trough. The first four major OP wavelets were included for analysis.

### Optical coherence tomography (OCT)

Spectral domain-optical coherence tomography (SD-OCT), which is emerging as a reliable tool for non-invasively evaluating structural changes in the retina in both clinical and research fields [27–29], was conducted as an in-vivo assessment of retinal morphology. Change of retinal thickness, measured by OCT, was used to reflect the extent of cell degeneration. The experimental time points and preparation procedures, including anesthetization and pupil dilation, were the same as those described for the ERG measurement. To maximize the image quality, the cornea was maintained moist with lubricant containing 0.3% propylene glycol and 0.1% polyethylene glycol 400 (Systane® ultra, Alcon) throughout the measurement.

OCT was performed using Bioptigen Envisu™ SD-OCT (R2210, Leica Microsystems, Morrisville, NC, USA). OCT rectangular scans (a-scans/b-scans: 1000 lines; b-scans: 100 scans; frame/b-scans: 10 frames) that covered an area of 0.8 × 0.8 mm were performed. The optic disc was located at the center of the scan and served as a reference point to ensure the scanning location was the same across animals and between different time points. The retinal thickness was analyzed by the built-in software InVivoVue Diver (v.3.0.8, Bioptigen Inc., Morrisville, NC, USA).

### Evaluation of retinal histology

The retinas of the mice in the two groups were processed for immunohistochemistry (IHC) and 4′,6-diamidino-2-phenylindole (DAPI) staining according to the procedures described previously [30]. At 25 weeks, the animals were euthanized by cervical dislocation. One eye from each animal (randomly chosen) was enucleated and dissected to isolate the whole retina, which was then incubated in 4% paraformaldehyde in phosphate buffer at room temperature for 1 h for fixation. The whole retinas were processed into vertical retinal sections (35 μm) using a microtome (Vibratome VT1200S, Leica Microsystems, Deer Park, IL, USA). The samples were then blocked with 10% donkey serum in TBS (0.5% Triton X-100 and 0.1% Sodium Azide in Dulbecco's phosphate-buffered saline, pH7.2) at 4 °C overnight to reduce non-specific labelling.

The samples were incubated with primary antibodies (anti-GNAT2: LifeSpan BioScience, Seattle, WA, USA; LS-C321680, dilution 1:75; anti-PKCα: Santa Cruz Biotechnology, Dallas, TX, USA; sc-8393, dilution 1:50; anti-bassoon: Cell Signaling Technology, Danvers, MA, USA; D63B6, dilution: 1:200) at 4 °C in TBS with 3% donkey serum for four days. Following incubation, the samples were washed several times and transferred to a 3% normal donkey serum-TBS solution containing donkey-host secondary antibodies conjugated with Cy3 (Millipore Sigma, Burlington, MA, USA, AP192C, dilution 1:200) or Alexa Fluor 488 (Invitrogen, A21206, dilution 1:1000) at 4 °C overnight. DAPI (Invitrogen, D1306) was used to stain the nuclei in the retina to evaluate the extent of neural cell survival by quantifying the number of viable cells.

A confocal laser scanning microscope (LSM800, Zeiss, Oberkochen, Germany) was used to capture the confocal micrographs of the specimens using a 20×, 40×, or 63× objective. To measure the number of cone photoreceptors and rod bipolar cells (and their synaptic terminals), GNAT2 and PKCα positive cells were counted in the whole imaged area (i.e., a 160 μm-segment), respectively, with a cell counter in ImageJ (v.1.53k, National Institutes of Health, Bethesda, MD, USA). To determine the number of photoreceptor cells (including both rods and cones), the DAPI stained nuclei in the outer nuclear layer were counted in half of the image area (an 80 μm-segment). For the number of rod-bipolar dendritic boutons, the number of PKCα positive puncta in the out plexiform layer was counted over a 20 μm-segment. The dimensions of the outer nuclear layer and the inner nuclear layer were analyzed by ImageJ. The data of each individual animal was averaged from three retinal sections, with each group consisting of at least five animals.

### Measurement of retinal mitochondrial bioenergetics

The mitochondrial bioenergetics of the retina of the db/db mice and db/+ mice were assessed by measuring the oxygen consumption rate (OCR) using the Seahorse XFe24 Extracellular Flux Analyzer (Agilent Technologies, Santa Clara, CA, USA) with reference to the protocol described by Millman et al. [31]. The Seahorse XF Analyzer evaluates the mitochondrial function of cells or tissues by serially injecting several chemicals that target the electron transport chain complexes throughout the assay to derive different key parameters of mitochondrial function.

One eye from each animal was enucleated and dissected to harvest the whole-mount retina in ice-cold PBS. Three 1.5 mm diameter punches were obtained from the neural retina with a biopsy puncher (Miltes Instrument, Integra LifeSciences, Mansfield, MA, USA). Each retinal punch, obtained adjacent to the optic nerve head to minimize variation in cell density, was carefully placed in the well of an XF24 Islet capture microplate (Agilent Technologies) with the ganglion cell layer facing up and covered with Islet Fluxpak mesh inserts (Agilent Technologies). Prior to the measurement of OCR, Seahorse XF DMEM medium (103335-100, Agilent Technologies) containing 5.5 mM glucose (G6152, Sigma-Aldrich, St Louis, MS, USA) and 1 mM sodium pyruvate (11360070, Thermo-Fisher, Waltham, MA, USA) was added to each well, and the retinal punches were incubated at 37 °C in a non-CO_2_ incubator for 60 min to allow the temperature and pH to reach equilibrium. The OCR was then measured under basal conditions and after serial injection of 1 µM carbonylcyanide-4-(trifluoro-methoxy) phenylhydrazone (FCCP; 15218, Cayman Chemical Company, Ann Arbor, Michigan, USA)), and a mixture of 10 µM rotenone (13995, Cayman) and 20 µM antimycin A (A8674, Sigma-Aldrich) to determine the values for basal respiration, maximal respiration, spare respiratory capacity, and non-mitochondrial oxygen consumption. After the assays, the retinal punches in each well were lysed with EB2 lysis buffer, containing 7 M urea, 2 M thiourea, 30 mM Tris, 2% (w/v) CHAPS, and 1% (w/v) ASB14 with protease inhibitor cocktail (Roche Applied Science, Basel, Switzerland), and placed on ice. The protein concentrations were determined using the Bradford Protein Assay (Bio-Rad Laboratories Inc., Hercules, CA, USA) according to the manufacturer’s guidelines for normalization of the OCR values.

### Statistical analysis

Data are presented as mean ± SEM. Shapiro–Wilk’s test was used to check the normality of data distribution prior to the use of a parametric test. For longitudinal comparison, mixed-model ANOVA was used to analyze the within-group, between-group, and interactive effects. Independent sample t-test (with Welch correction when appropriate) was used for comparison between the two groups. JASP (JASP Team, 2022, v.0.16.3) Amsterdam, Netherlands) was used for the statistical analysis. Differences with a *P* value less than 0.05 were considered significant.

## Results

### Metabolic characterization of the diabetic mice

**Fig. 1:**
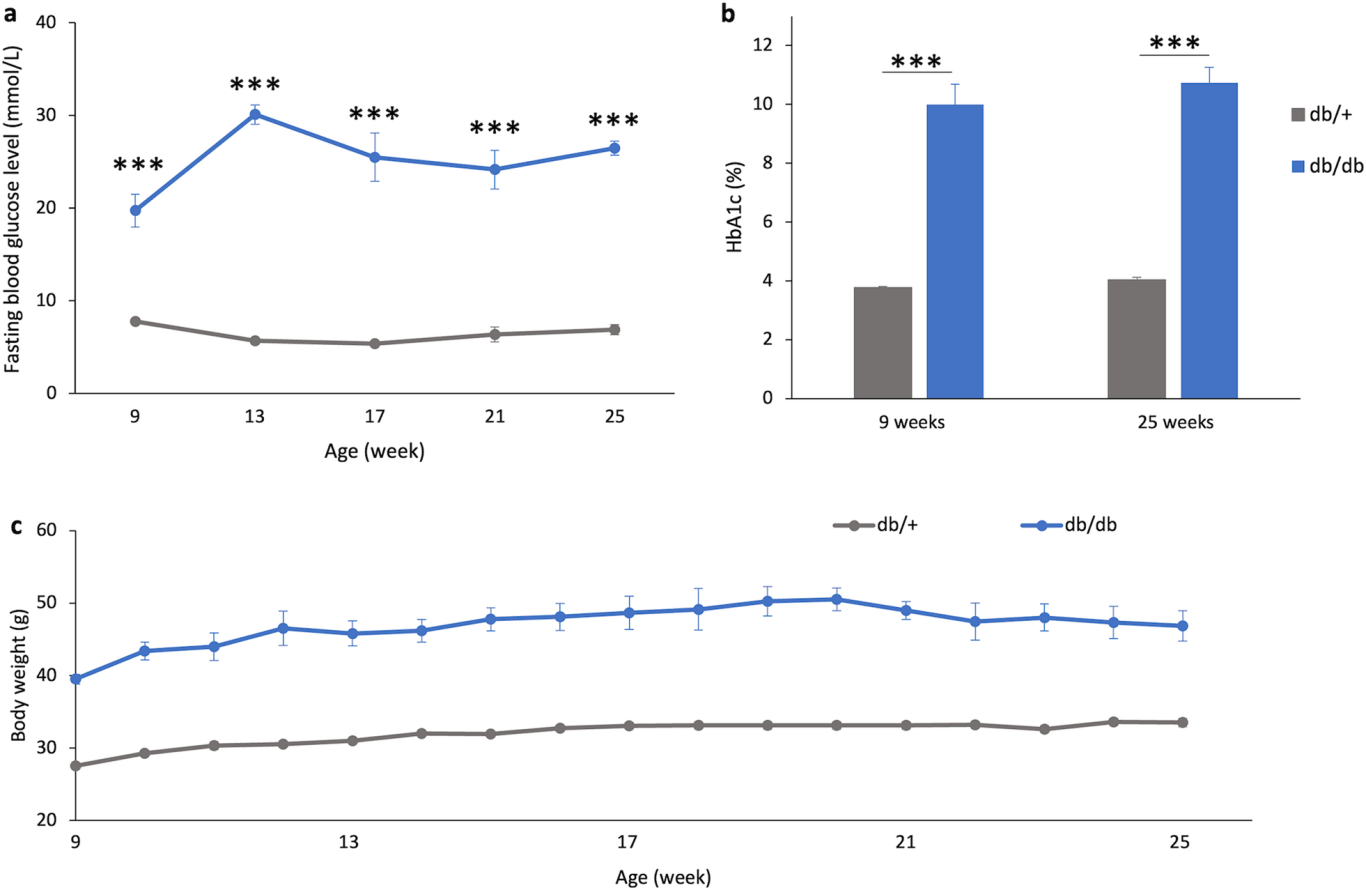
Diabetic-related indices of the two groups of mice. **a** Fasting blood glucose levels; **b** HbA_1c_ levels; **c** Body weight of the db/+ mice (n = 14) and db/db mice (n = 11). Data presented as mean ± SEM. Simple main effect analysis: ***P < 0.001, db/ + mice vs. db/db mice

In agreement with earlier reports, the fasting blood glucose levels of db/db mice at 9 weeks of age were higher than 13.9 mmol/L, reaching the levels of established diabetes. With time, the db/db mice continued to display higher fasting blood glucose levels than db/+ mice (Fig. 1a; repeated measures ANOVA (Greenhouse–Geisser corrected), time: F = 5.130, df = 2.771, *P* = 0.004; group: F = 324.128, df = 1, *P* < 0.001; time*group: F = 9.913, df = 2.771, *P* < 0.001). In addition, the HbA_1c_ levels, which reflect the overall glycemic levels over a three-to-four-month period, were also significantly higher in db/db mice than db/+ mice (Fig. 1b) at 9 weeks (db/+ mice: 3.74 ± 0.04% vs. db/db mice: 8.66 ± 0.62%; Simple main effect analysis: F = 80.006, df = 1, *P* < 0.001) and 25 weeks (db/+  mice: 4.04 ± 0.08% vs. db/db mice: 11.14 ± 0.42%; Simple main effect analysis: F = 322.522, df = 1, *P* < 0.001).

Consistent with the reported diabetic phenotype of this mouse model, the db/db mice were significantly heavier than their normoglycemic heterozygous littermates (db/+ mice) at 9 weeks and throughout the experimental period (Fig. 1c). A mixed model ANOVA revealed a significant time (F = 7.021, df = 16, *P* < 0.001) and group effect (F = 86.880, df = 1, *P* < 0.001), with the interaction also being significant (F = 9.297, df = 16, *P* < 0.01). The db/db mice also consumed more food and water when compared to db/+ mice (Additional file 1: Table S1).

### Decline of inner retinal function in db/db mice

**Fig. 2:**
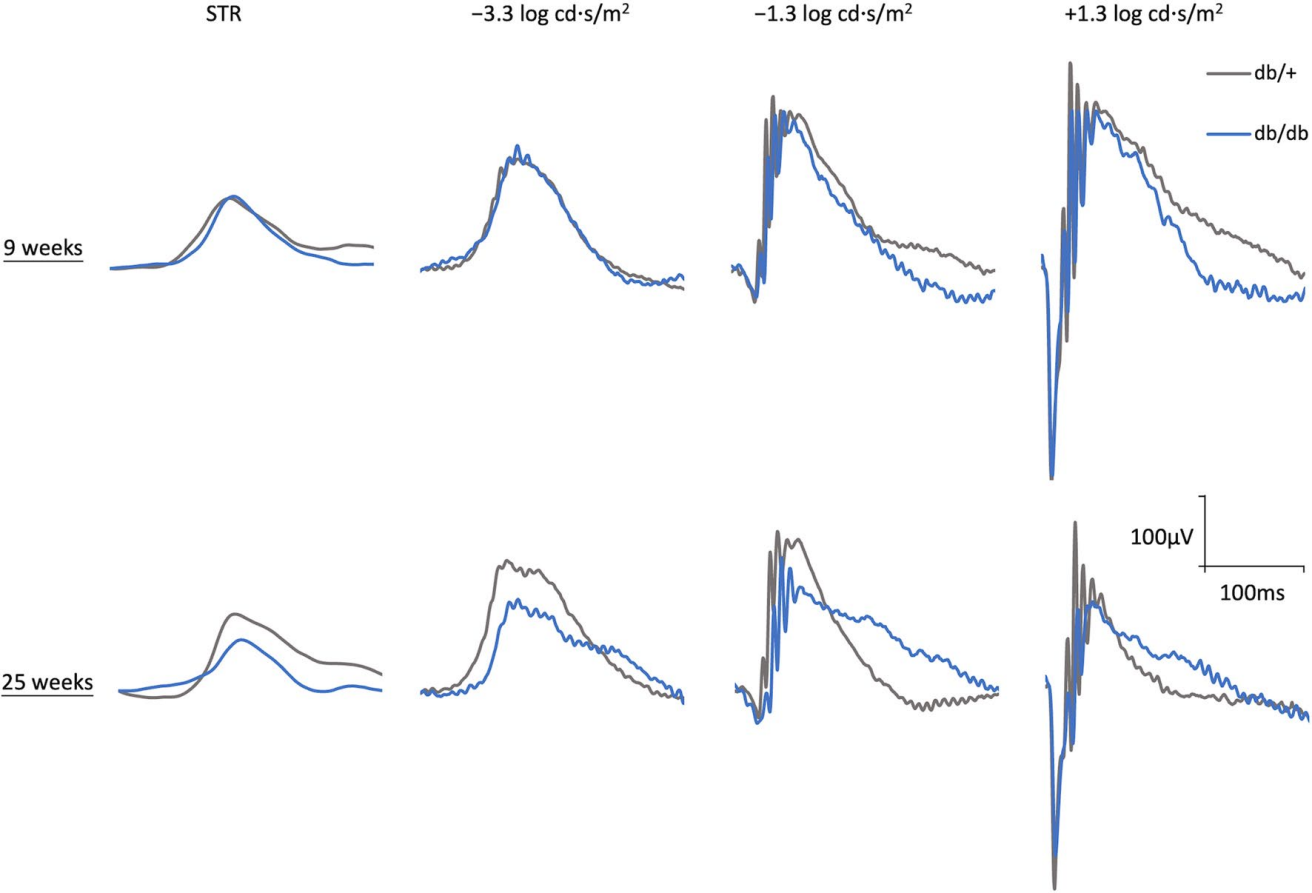
Representative raw electroretinography (ERG) waveforms. The representative raw ERG waveforms of db/+ mice (grey) and db/db mice (blue) recorded at 9 and 25 weeks of age at four different stimulus intensities belonging to four different light zones, as described previously [25]. The scotopic threshold response (STR) refers to the response from the first light zone, which is suggested to arise from third-order retinal cells, including ganglion cells and amacrine cells. Responses from the second light zone (i.e., − 3.3 log cd·s/m^2^) represent predominantly rods to rod-depolarizing bipolar cells responses, while those from the third light zone (i.e., − 1.3 log cd·s/m^2^) consist of responses from rods to rod-depolarizing bipolar cells and rods to cone-depolarizing bipolar cells. In addition, the responses from the fourth light zone (i.e., + 1.3 log cd·s/m^2^) involve signals from the cone-related pathway

The retinal function was assessed by ffERG, a technique that measures the mass electrical response of retinal cells (including neuronal and glial cells) to diffuse flashes of light to reflect retinal functions [32]. The different characteristics of various retinal cell types towards light leads to different components of the ERG waveform as elicited by stimuli with various intensities. The representative ERG waveforms of the two groups of mice at different timepoints are shown in Fig. 2.

The a-wave of scotopic ERG is attributed to the photoreceptor cells, in particular, the rod cells. From 9 to 25 weeks of age, the differences in both the amplitude (Additional file 1: Fig. S1a–d) and implicit time (Additional file 1: Fig. S1e–h) of scotopic a-waves did not differ significantly between the db/db mice and db/+ mice. The results suggested that photoreceptor cell functions were not affected in this diabetic mouse model within the study period.

**Fig. 3:**
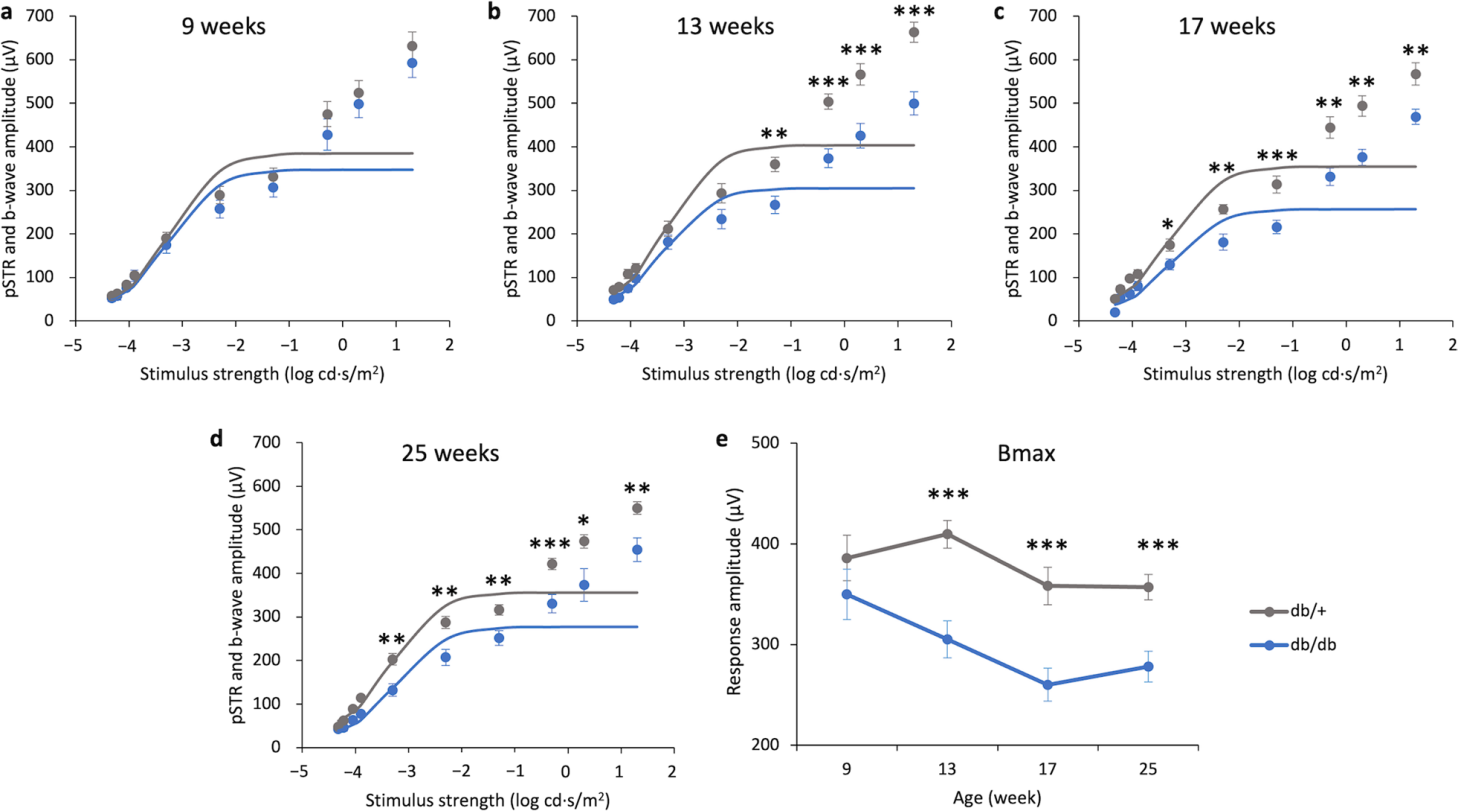
Electroretinography (ERG) signal declined with time in db/db mice. **a–d** Stimulus–response plots showing the amplitude of scotopic ERG pSTR and b-wave response recorded from db/+ mice (n = 14) and db/db mice (n = 11) at different experimental timepoints. The responses within the rod operative range (i.e., ≤  − 0.3 log cd·s/m^2^) were fitted with sigmoidal curves, using the Naka-Ruston function to deduce the maximum b-wave response (Bmax) value. **e** The Bmax value of the two groups of mice over the experimental period. Data presented as mean ± SEM. Simple main effect analysis: *P < 0.05, **P < 0.01, ***P < 0.001

At 9 weeks, the b-wave responses did not differ significantly between db/db mice and db/+ mice. In contrast to the a-wave, the b-wave amplitude started to decline with increased duration of diabetes (Fig. 3a–e). At 25 weeks, the b-wave amplitude of db/db mice at all tested stimulus intensities was significantly reduced compared to their non-diabetic littermates. To determine the rod-driven b-wave maximum response amplitude, the ERG responses within the rod operation range were fitted with sigmoidal curves using the Naka-Ruston function, a saturating hyperbolic function that describes the relationship between b-wave amplitude and flash intensity [25]. The Bmax value deduced from this function was used for analysis. Repeated measures ANOVA revealed a significant main effect of time and groups (repeated measures ANOVA, time: F = 6.407, df = 3, *P* < 0.001; group: F = 22.663, df = 1, *P* < 0.001; time × group: F = 1.824, df = 3, *P* = 0.151). At 9 weeks, the difference in the Bmax value was not statistically significant between the two groups of mice. However, the Bmax value of db/db mice became significantly lower than that of the db/+ mice at the later time points (i.e., 13 to 25 weeks of age), indicating that the rod-related pathway function had been affected progressively over time (Fig. 3f). In addition, the implicit time of the b-wave showed a tendency of delay in db/db mice at some tested stimulus intensities (Additional file 1: Fig. S2) compared to the db/+ mice.

**Fig. 4:**
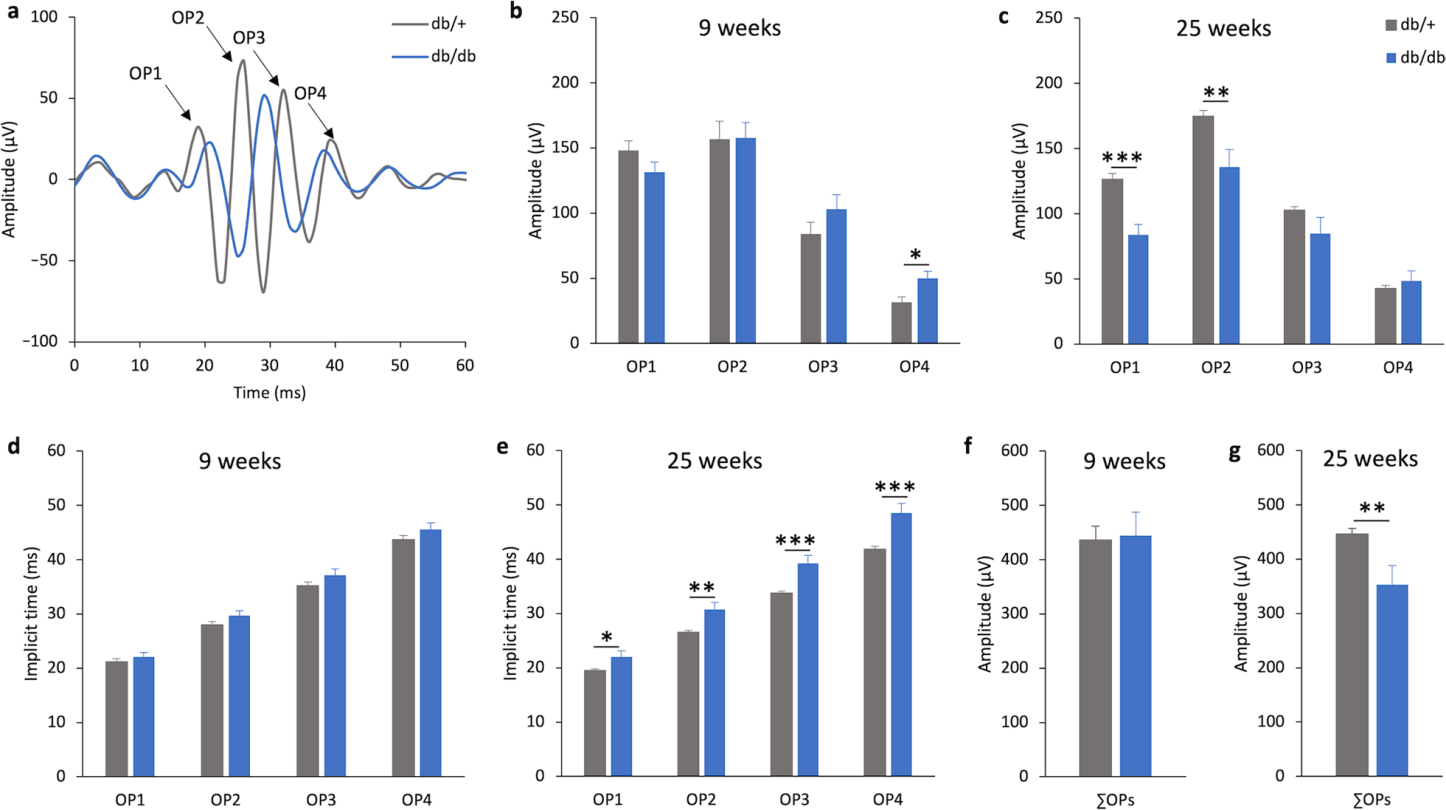
Reduced amplitude and delayed implicit time of oscillatory potentials (OPs) were found in db/db mice. **a** The representative OPs waveforms recorded at + 0.3 log cd·s/m^2^ of db/+ (grey) and db/db (blue) mice at 25 weeks of age. Arrows indicating the peaks of OP1 to OP4 of the db/+ mice. **b–e** Bar charts showing the mean amplitudes and implicit times of OP1 to OP4 and of the db/+ mice (n = 14) and db/db mice (n = 11) at 9 and 25 weeks. Data presented as mean ± SEM. Independent sample t-test: *P < 0.05, **P < 0.01, ***P < 0.001

OPs, which are mainly associated with the amacrine cells, have been suggested to be an indicator of inner retinal function [33]. Digital filtering of the ERG response at + 0.3 log cd·s/m^2^ was used to obtain the OPs. Representative waveforms of the OPs obtained at 25 weeks are shown in Fig. 4a, with arrows indicating the peak of the OPs of db/+ mice. At 9 weeks, the implicit time of the four major OPs and the amplitude of OP1 to OP3 and the ∑OPs were not statistically different between the two groups of mice (Fig. 4b, d and f). However, the amplitude of OP4 of db/db mice was significantly stronger than that of the db/+ mice (db/+  = 31.80 ± 4.00 μV vs. db/db = 49.89 ± 5.56 μV; Independent sample t-test, t = 2.709, df = 23, *P* = 0.013). The results showed that the amplitudes of the first two major OP wavelets (Fig. 4c, OP1: db/+  = 126.31 ± 4.42 μV vs. db/db = 83.89 ± 7.92 μV; Independent sample t-test, t =  − 4.939, df = 23, *P* < 0.001; OP2: db/+  = 174.63 ± 4.53 μV vs. db/db = 135.81 ± 13.44 μV; Independent sample t-test, t =  − 3.008, df = 23, *P* = 0.006), as well as the ∑OPs amplitude (Fig. 4g, db/+  = 446.37 ± 10.24 μV vs. db/db = 353.10 ± 34.77 μV; Independent sample t-test, t =  − 2.847, df = 23, *P* = 0.009), were significantly reduced in db/db mice compared to db/+ mice at the later timepoint (i.e., 25 weeks of age). The implicit times of all OPs of db/db mice were also significantly delayed compared to their normoglycemic littermates (Fig. 4d).

Moreover, the study results showed that the implicit time and amplitude of positive scotopic threshold response (pSTR, response presumably originated from the ganglion cells [25]) did not differ statistically between db/db mice and db/+ mice at 9 weeks (Additional file 1: Fig. S3a–d), although with time, decline in the amplitude of pSTR at some of the tested intensities was observed. Similarly, the pSTR implicit time was found to be similar between the diabetic and control mice at 9 and 13 weeks, but that of the db/db mice was delayed at some of the tested intensities at 17 and 25 weeks (Additional file 1: Fig. S3e–h).

### Reduced retinal thickness early after diabetes onset

**Fig. 5:**
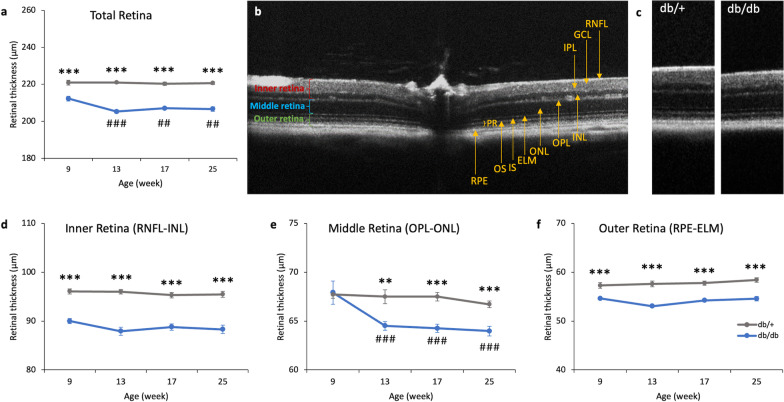
Longitudinal profiles of retinal thickness measured with OCT revealed thinner retinas in db/db mice. **a** Line charts showing the mean total retinal thickness of db/+ mice (n = 14) and db/db mice (n = 11) at different experimental timepoints. **b** A b-scan tomogram from an equatorial slice of the scan showing the different sublayers in db/+ mice. **c** Representative OCT images showing a retinal segment approximately 118 μm away from the center of optic nerve head of db/+ and db/db mice at 25 weeks of age. Line charts showing the mean thickness of the **d** inner retina, **e** middle retina, and **f** outer retina of the two groups of mice at different experimental timepoints. Data presented as mean ± SEM. Simple main effect analysis: **P < 0.01, ***P < 0.001 vs*.* db/db mice; ^##^*P* < 0.01, ^###^*P* < 0.001 vs. db/db at 9 weeks. RNFL, retinal nerve fiber layer; GCL, ganglion cell layer; IPL, inner plexiform layer; INL, inner nuclear layer; OPL, outer plexiform layer; ONL, outer nuclear layer; ELM, external limiting membrane; OS, outer segment; IS, inner segment; PR, photoreceptor; RPE, retinal pigmented epithelium

To investigate the structural changes of the retina over the course of diabetes, the retinal thickness was assessed longitudinally using SD-OCT. The results showed a significant main effect of time and group, as well as a significant interaction between time and groups in the total retinal thickness (repeated measures ANOVA, time: F = 6.251, df = 1.935, *P* < 0.004; group: F = 198.702, df = 1, *P* < 0.001; time × group: F = 5.824, df = 1.935, *P* = 0.006). The total retinal thickness was significantly thinner in db/db mice than db/+ mice at all experimental timepoints (Fig. 5a). In addition, the total retinal thickness of db/db mice was found to be significantly reduced from 13 to 25 weeks compared to the measurement at 9 weeks. The changes in retinal thickness were further assessed by dividing the retina into three portions: inner [retinal nerve fiber layer (RNFL)-inner nuclear layer (INL)], middle [outer plexiform layer (OPL)-outer nuclear layer (ONL)], and outer [retinal pigment epithelium (RPE)-external limiting membrane (ELM)] retina (as illustrated in Fig. 5b). The inner and outer retinas of the db/db mice were significantly thinner than those of the db/+ mice throughout the experimental period (Fig. 5d & f). However, the middle retina thickness was found to be similar in the two groups of mice at 9 weeks (Fig. 5e), only starting to decrease at 13 weeks, becoming significantly thinner in db/db mice than db/+ mice (repeated measures ANOVA, time: F = 7.551, df = 2.225, *P* < 0.001; group: F = 19.936, df = 1, *P* < 0.001; time × group: F = 4.212, df = 2.225, *P* = 0.017).

### Changes in retinal histology

**Fig. 6:**
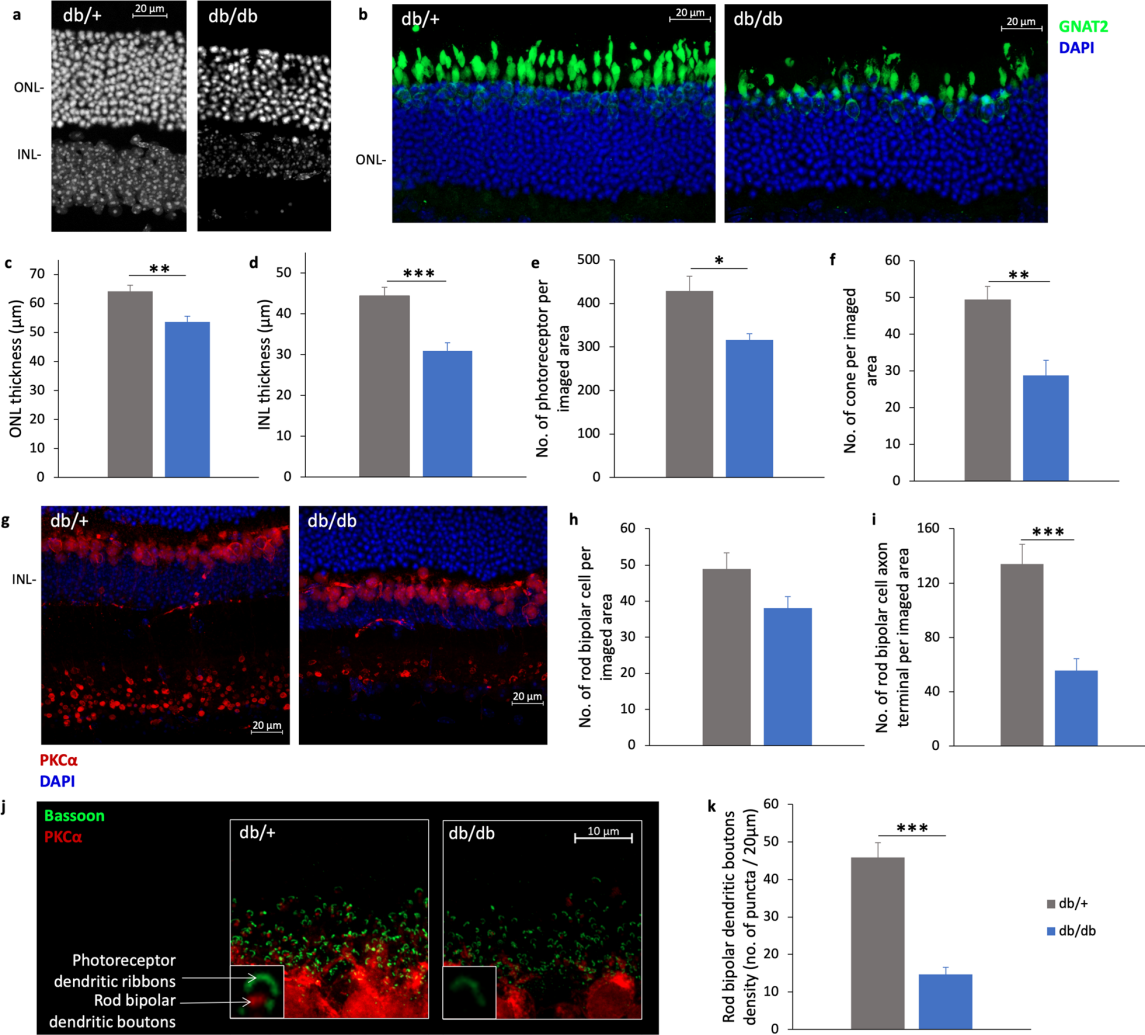
Changes in retinal histology revealed in db/db mice. **a**, **b** Representative confocal images of the retinal sections of the two groups of mice processed using 4′,6-diamidino-2-phenylindole (DAPI, white or blue) and G Protein Subunit Alpha Transducin 2 (GNAT2, green). White scale bar: 20 μm. **c–f** Bar charts comparing the thickness of the outer nuclear layer (ONL) and inner nuclear layer (INL), the number of photoreceptors (DAPI stained nuclei in the ONL), and the number of cones (soma co-labelled by GNAT2) of db/+ mice (n = 7) and db/db mice (n = 6). (Data presented as mean ± SEM. Independent sample t-test: *P < 0.05, **P < 0.01, ***P < 0.001.) Representative confocal images of the retina sections of db/+ mice and db/db mice processed using PKCα (red) and DAPI (blue). White scale bar: 20 μm. **h**, **i** Bar charts comparing the rod bipolar cell densities and number of rod bipolar cell axon terminals of db/+ mice (n = 7) and db/db mice (n = 6). **j** Representative confocal images of retinal sections of db/+ mice and db/db mice stained with PKCα (red) and bassoon antibodies (green). White scale bar: 10 μm. **k** Bar charts comparing the rod bipolar cell dendritic boutons of db/+ mice (n = 7) and db/db mice (n = 5). Data presented as mean ± SEM. Independent sample t-test: ***P < 0.001

At 25 weeks of age, one eye from each animal was removed for sectioning and IHC analysis. In agreement with the OCT findings, the thickness of both ONL and INL were significantly reduced in the retina of db/db mice compared to db/ + mice (Fig. 6a, c, d; ONL: db/+  = 65.78 ± 2.72 μm vs. db/db = 53.60 ± 2.02 μm; Independent sample t-test: t =  − 3.488, df = 11, *P* = 0.005; INL: db/+  = 44.40 ± 1.93 μm vs. db/db = 30.85 ± 1.55 μm; Independent sample t-test: t =  − 5.347, df = 11, *P* < 0.001). The density of the photoreceptor cells was evaluated by counting the DAPI-stained cells in the ONL over an 80 μm-segment [1 segment/retinal section; 3 retinal sections/animal; n = 7(db/+)/6(db/db)], which revealed that the photoreceptor cell density was significantly lower in db/db mice than db/+ mice (Fig. 6b and e; db/+  = 428.32 ± 33.82 vs. db/db = 316.41 ± 14.35; Independent sample t-test: t =  − 2.865, df = 11, *P* = 0.015). In addition, db/db mice had significantly fewer cone soma (nucleus co-stained with GNAT2) than db/+ mice (Fig. 6f; db/+  = 49.41 ± 3.58 vs. db/db = 28.76 ± 4.10; Independent sample t-test: t =  − 3.815, df = 11, *P* = 0.003).

As the scotopic ERG result showed a significant reduction in Bmax value in db/db mice as early as 13 weeks of age, immunostaining of the rod-bipolar cells with antibodies against PKCα was performed to determine if these cells were affected. Representative confocal images of the retinal sections of the two groups of mice are shown in Fig. 6g. Although the rod bipolar cell density tended to be lower in db/db mice, the difference did not reach statistical significance (Fig. 6h; db/+  = 48.91 ± 4.17 vs. db/db = 38.01 ± 3.20; Independent sample t-test: t =  − 1.933, df = 11, *P* = 0.079). However, the number of rod-bipolar cell axon terminals (Fig. 6i) was significantly reduced in db/db mice compared to db/+ mice (db/+  = 134.21 ± 12.13 vs. db/db = 55.75 ± 8.75; Independent sample t-test: t =  − 5.080, df = 11, *P* < 0.001). The photoreceptor synaptic ribbons were co-stained with antibodies against bassoon, which revealed absence of paired connectivity between photoreceptor terminals and second-order neurons was more prominent in db/db mice than db/+ mice (Fig. 6j). The number of rod-bipolar cell dendritic boutons (Fig. 6k) was also significantly reduced in db/db mice compared to db/+ mice (db/+  = 45.88 ± 3.87 vs. db/db = 14.69 ± 1.91; Independent sample t-test: t =  − 6.356, df = 10, *P* < 0.001).

### Retinal mitochondrial bioenergetics of diabetic mice

**Fig. 7:**
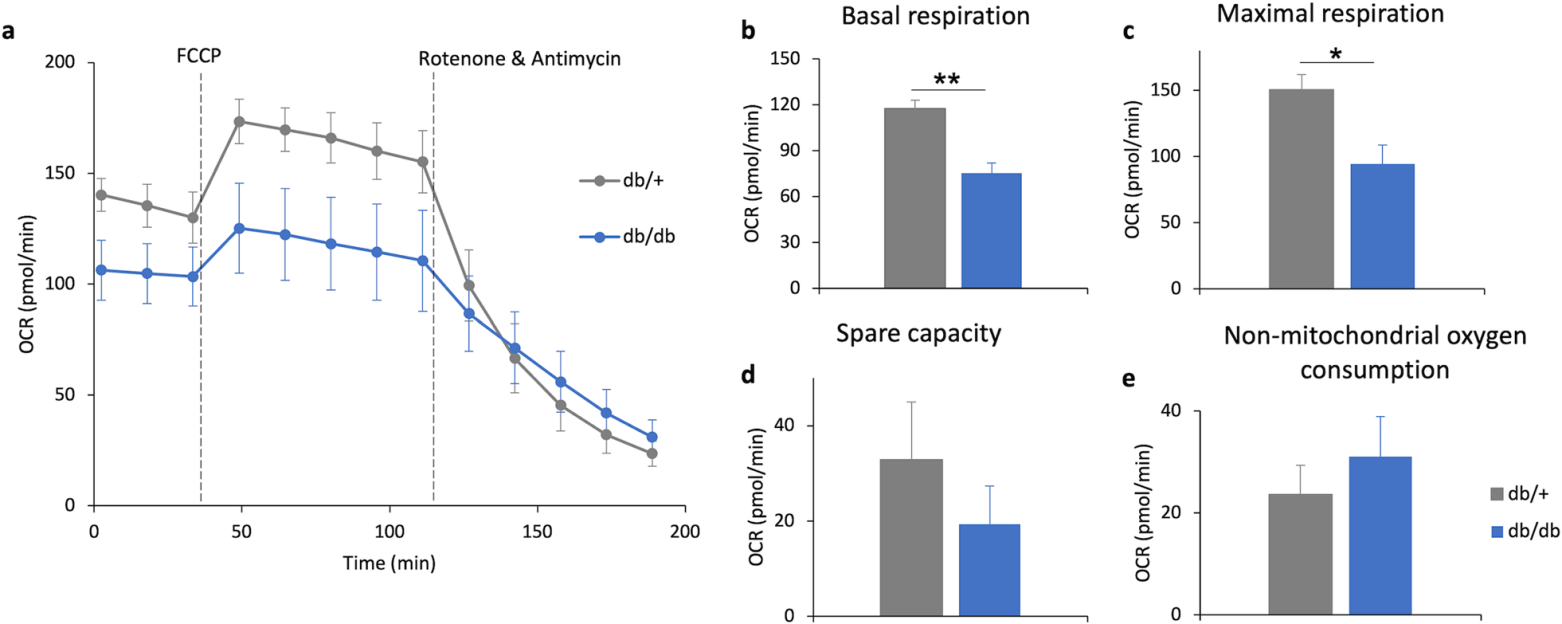
Diabetes led to reduced retinal mitochondrial bioenergetic in db/db mice. **a** The representative oxygen consumption rate (OCR) measured from the two groups of mice. **b** Graphs showing the key mitochondrial function parameters of db/+ (n = 5) and db/db (n = 7) mice. Data presented as means ± SEM. Unpaired sample t-test, *P < 0.05; **P < 0.01. FCCP: carbonyl cyanide 4-(trifluoromethoxy) phenylhydrazone

The mitochondrial bioenergetics on the ex vivo retina of the db/db mice and db/+ mice at 25 weeks of age were tested using a Seahorse XF Analyzer (Fig. 7). Compared to the db/+ mice, db/db mice exhibited significantly lower basal respiration (db/+  = 122.76 ± 7.93 pmol/min vs. db/db = 75.29 ± 6.75 pmol/min; Independent sample t-test: t =  − 4.554, df = 10, *P* = 0.001) and maximal respiration (db/+  = 147.87 ± 11.98 pmol/min vs. db/db = 94.41 ± 14.11 pmol/min; Independent sample t-test: t =  − 2.725, df = 10, *P* = 0.021). These results indicate compromised mitochondrial function in the retina of db/db mice compared to db/ + mice.

## Discussion

The results of this study provide longitudinal evidence that neurodegeneration occurs in the retina of db/db mice in the early stage of diabetes, in which the ERG signals were decreased with neurosensory layers in the retina thinned and the photoreceptor and cone density reduced. In agreement with the results of other studies [9, 34–36], reduced amplitude and delayed implicit time in OPs and pSTR were observed in the db/db mice compared to the db/+ mice, indicating that diabetes leads to compromised inner retinal function. Neuroretinal changes have been suggested as one of the earliest manifestations of DR [8–12]. Recent studies have revealed that neuronal activity could modulate glial function by affecting ion transport signaling and coupling ratio of astrocytes and microglia, via activation of various receptors of neuromodulators, including but not limited to acetylcholine, noradrenaline, and dopamine [37–40]. Furthermore, neuronal stress/death can trigger reactive gliosis [41, 42], which in turn disrupts retinal homeostasis and barrier integrity, aggravating the development of DR.

Intriguingly, although the scotopic ERG a-wave did not differ statistically between the db/db mice and the db/+ mice, the ERG result did reveal a reduced Bmax value in db/db mice compared to db/+ mice, indicating a diminished function of the rod photoreceptor and/or its related postsynaptic pathway. It was unexpected that the number of rod-bipolar cells was not significantly lower in the db/db mice compared to the db/+ mice. However, upon studying the synaptic connections, it was observed that the number of rod-bipolar cell dendritic boutons and axon terminals was significantly reduced in the db/db mice relative to the db/+ mice, suggesting a compromised synaptic connectivity between the first- and the second-order neurons in the diabetic retina before their viability was affected. A similar disruption of synaptic structures in the second-order retinal neurons at the outer plexiform layer without apparent decreased bipolar cell viability has been demonstrated previously in Ins2^Akita^ mice following longer-term diabetes (6–9 months) [43]. The mechanism leading to this observed change in synaptic structure remains elusive, but is speculated to be caused by a Dynamin-1-like protein-dependent signaling that disrupts mitochondrial dynamics and reduces mitochondrial trafficking into dendrites, resulting in localized ATP deficiency, synaptic calcium dysregulation, and dendritic disintegration, as observed in cultured Purkinje cells [44]. The current study supports the role of mitochondrial dysfunction in the development of DR by showing reduced retinal mitochondrial function was accompanied by the electrophysiological and structural changes in db/db mice model.

Defective mitochondrial biogenesis and changes in energy metabolic profile have been documented in neurons, myocytes, and various tissue affected by type 2 diabetes [45–47]. Mitochondrial dysfunction is suggested as a potential pathway contributing to the pathogenesis of DR and is observed in a range of retinal cells under simulated hyperglycemia [13–15]. Our group has recently demonstrated that high glucose compromises the mitochondrial function of the 661W photoreceptor-like cell line [16]. However, how diabetes affects mitochondrial function in retinal tissue is not well understood. In light of this, the current study investigated whether diabetes affected the mitochondrial function of the retina in the db/db mouse model ex vivo using the Seahorse XF analyzer. The results showed that diabetes leads to decreased basal respiration and maximal respiration in the retina of db/db mice compared to their non-diabetic littermates.

The primary driver leading to the diminished mitochondrial function in the retina of db/db mice is not explicit, and several potential pathways may lead to compromised mitochondrial function and neurodegeneration in DR. One of the most studied is the dopaminergic pathway. Dopamine is a critical neurotransmitter mediating visual function in the retina [48, 49], and diminished dopamine levels have been observed in the retina of diabetic mice [50]. Use of levodopa, a precursor to dopamine, has been shown to alleviate high glucose-induced retinal microvascular leakage and abnormalities by reducing oxidative stress and mitochondrial dysfunction [51]. Nevertheless, together with other studies, the current study indicated that retinal neurodegeneration may occur in early DR development due to mitochondrial dysfunction. Increased oxidative stress and mitochondrial reactive oxygen species production have been demonstrated in the diabetic retina [52, 53]. By reducing oxidative damage and enhancing the quality control machinery of mitochondria, therapeutic approaches, such as using resveratrol and bromocriptine (a dopamine receptor D2 agonist), appear to be promising interventions to halt the development and progression of DR [54–57].

## Conclusion

The current study provided longitudinal evidence that db/db mice undergo neurodegeneration during the early development of diabetes, which induced structural and functional deterioration in the neuroretina of db/db mice. A decline in mitochondrial function was also demonstrated in the ex vivo retina of the db/db mice. It is noteworthy that these changes occurred before overt vasculopathy could be detected. Mitochondrial dysfunction has emerged as a critical mechanism contributing to the pathogenesis of DR, and the current study findings complement the growing body of literature that implicate mitochondrial dysfunction as an underlying cause triggering the development of DR. In addition, the db/db mouse model appears to be a useful animal model for testing potential treatment regimens targeting the early stages of DR.

## Supplementary Information

**Additional file 1: Table S1**. The average total food and water consumption of db/+ mice and db/db mice during the experimental period. **Figure S1.** Scotopic electroretinography (ERG) a-wave. **a**–**d** Stimulus–response plots showing the amplitude of scotopic ERG a-wave response recorded from db/ + mice (n = 14) and db/db mice (n = 11) at different experimental timepoints. **e**–**h** Bar charts comparing the implicit time of the ERG a-wave at different experimental timepoints. Data presented as mean ± SEM. Simple main effect analysis: * *P* < 0.05. SEM, standard error of the mean. **Figure S2.** Implicit times of scotopic electroretinography (ERG) b-wave. **a-d** Bar charts comparing the implicit times of the ERG b-waves from db/+ mice (n = 14) and db/db mice (n = 11) at different experimental timepoints. Data presented as mean ± SEM. Simple main effect analysis: *P < 0.05, **P < 0.01, ***P < 0.001. SEM, standard error of the mean. **Figure S3.** Positive scotopic threshold responses (pSTR). Bar charts comparing the amplitudes (**a–d**) and the implicit times (**e**–**h**) of the pSTR from db/ + mice (n = 14) and db/db mice (n = 11) at different experimental timepoints. Data presented as mean ± SEM. Simple main effect analysis: *P < 0.05, **P < 0.01, ***P < 0.001. SEM, standard error of the mean.

## Data Availability

The data presented in this study are available on request from the corresponding author.

## Abbreviations

DR: Diabetic retinopathy
ffERG: Full-field electroretinography
Bmax: Maximum b-wave response
OPs: Oscillatory potentials
STR: Scotopic negative response
FFT: Fast Fourier transform
OCT: Optical coherence tomography
ONL: Outer nuclear layer
INL: Inner nuclear layer
IHC: Immunohistochemistry
DAPI: 4′,6-diamidino-2-phenylindole
OCR: Oxygen consumption rate
