# Supplementary material for: Functional and structural changes in the neuroretina are accompanied by mitochondrial dysfunction in a type 2 diabetic mouse model
Source: Eye Vis (Lond). 2023 Sep 1;10:37. doi: 10.1186/s40662-023-00353-2 (PMC10472703; doi:10.1186/s40662-023-00353-2)

**Additional file**

**Table S1**. The average total food and water consumption of db/+ mice and db/db mice during the experimental period.

| Parameter |  | Mean ± SEM (g) | *P* value* |
| --- | --- | --- | --- |
| Total food consumption | db/+ | 629.94 ± 24.39 | 0.004 |
|  | db/db | 809.83 ± 99.93 |  |
| Total water consumption | db/+ | 673.08 ± 66.56 | <0.001 |
|  | db/db | 2167.83 ± 154.39 |  |

*SEM* = standard error of the mean. *: Independent sample t-test.

**Figure S1.** Scotopic electroretinography (ERG) a-wave. **a-d** Stimulus-response plots showing the amplitude of scotopic ERG a-wave response recorded from db/+ mice (n=14) and db/db mice (n=11) at different experimental timepoints. **e-h** Bar charts comparing the implicit time of the ERG a-wave at different experimental timepoints. Data presented as mean ± SEM. Simple main effect analysis: * *P*<0.05. SEM, standard error of the mean.


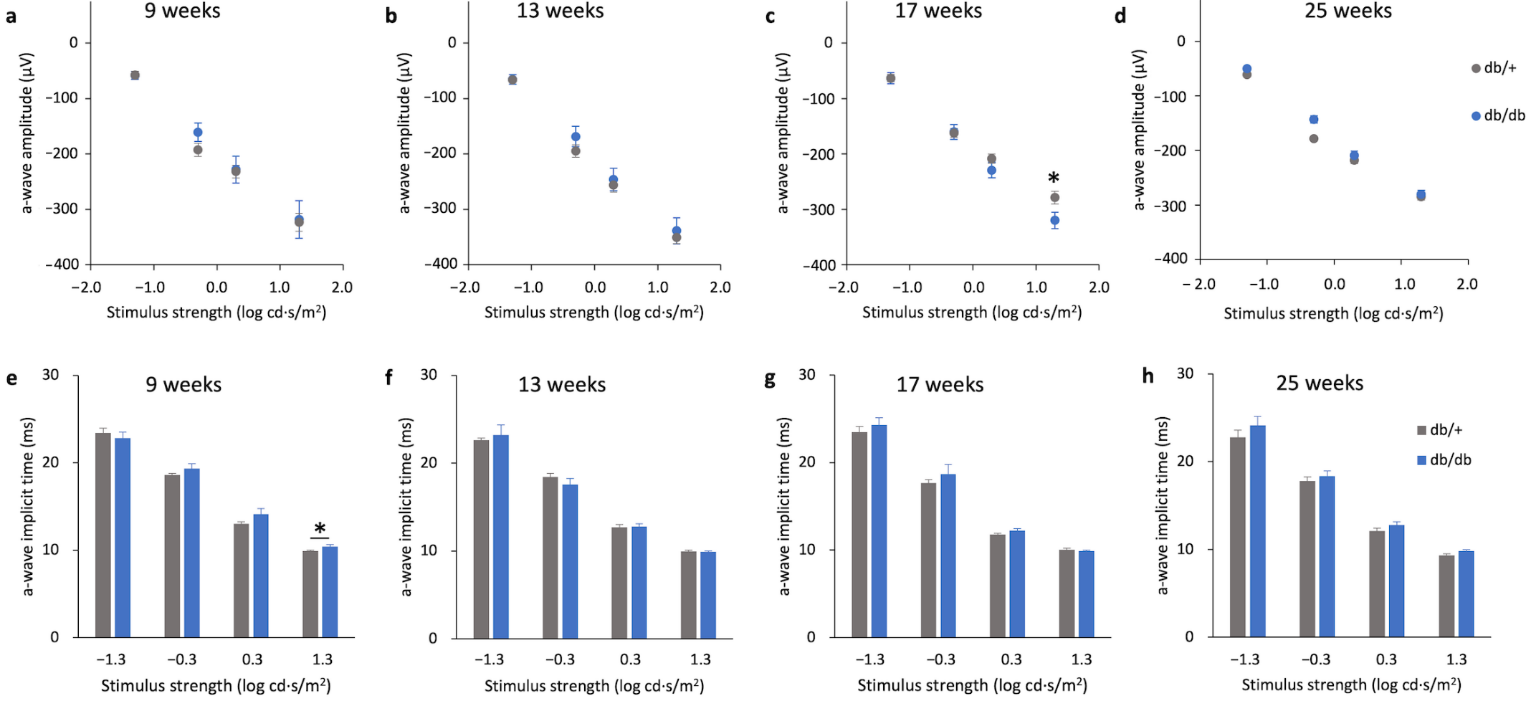


**Figure S2.** Implicit times of scotopic electroretinography (ERG) b-wave. **a-d** Bar charts comparing the implicit times of the ERG b-waves from db/+ mice (n=14) and db/db mice (n=11) at different experimental timepoints. Data presented as mean ± SEM. Simple main effect analysis: * *P*<0.05, ** *P*<0.01, *** *P*<0.001. SEM, standard error of the mean.


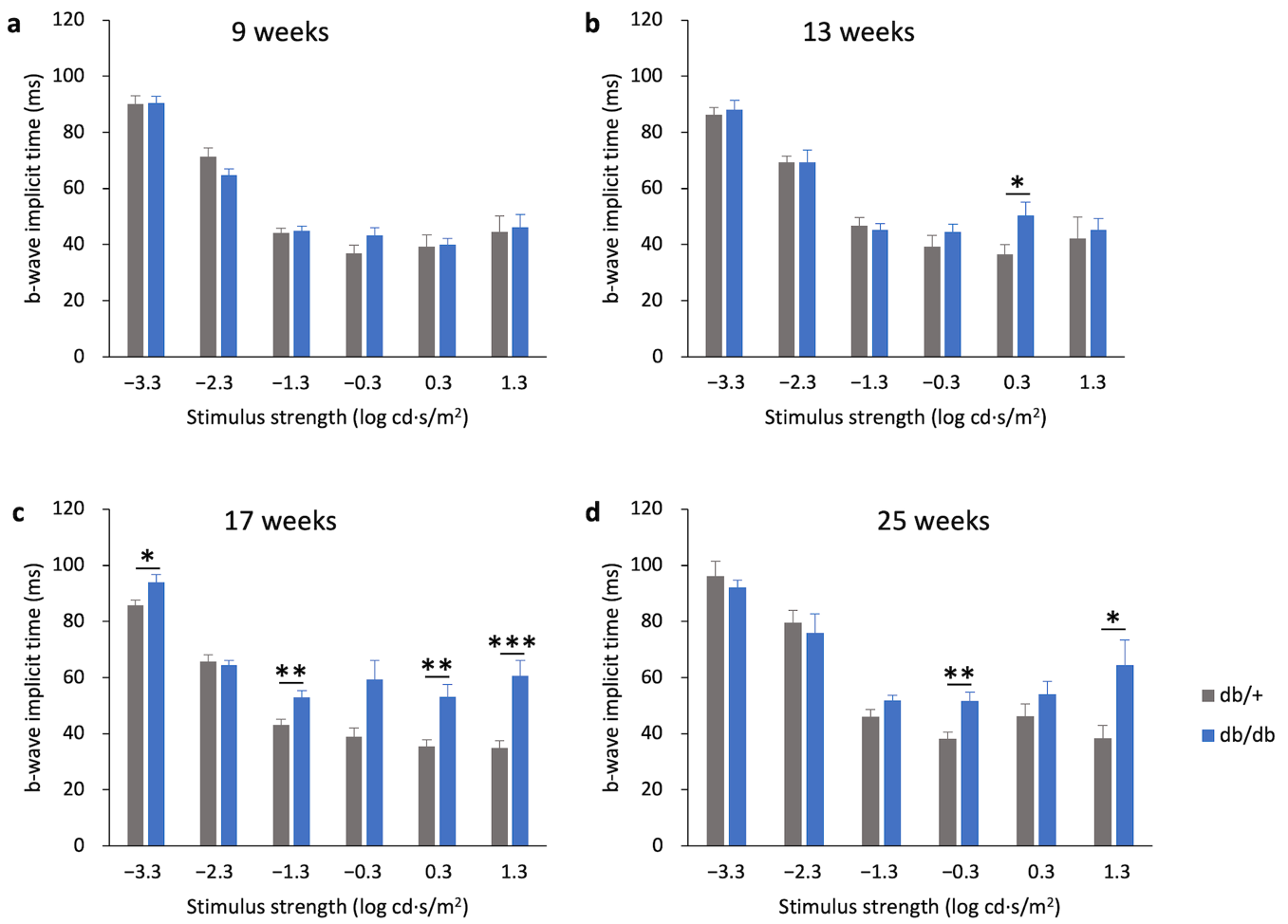


**Figure S3.** Positive scotopic threshold responses (pSTR). Bar charts comparing the amplitudes (a-d) and the implicit times (e-h) of the pSTR from db/+ mice (n= 14) and db/db mice (n=11) at different experimental timepoints. Data presented as mean ± SEM. Simple main effect analysis: * *P*<0.05, ** *P*<0.01, *** *P*<0.001. SEM, standard error of the mean.


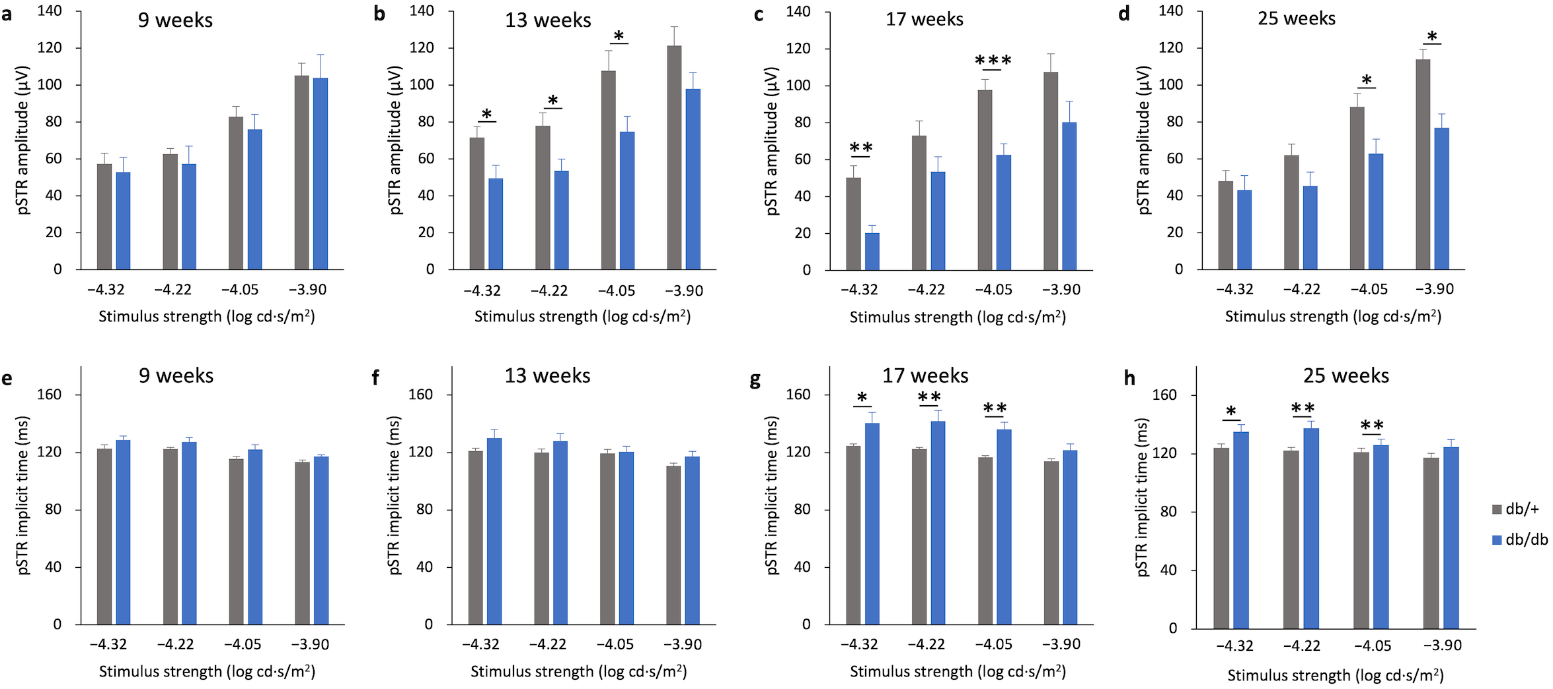

Supplement: Supplementary file 1 — Additional file 1: Table S1. The average total food and water consumption of db/+ mice and db/db mice during the experimental period. Figure S1. Scotopic electroretinography (ERG) a-wave. a–d Stimulus–response plots showing the amplitude of scotopic ERG a-wave response recorded from db/ + mice (n = 14) and db/db mice (n = 11) at different experimental timepoints. e–h Bar charts comparing the implicit time of the ERG a-wave at different experimental timepoints. Data presented as mean ± SEM. Simple main effect analysis: * P < 0.05. SEM, standard error of the mean. Figure S2. Implicit times of scotopic electroretinography (ERG) b-wave. a-d Bar charts comparing the implicit times of the ERG b-waves from db/+ mice (n = 14) and db/db mice (n = 11) at different experimental timepoints. Data presented as mean ± SEM. Simple main effect analysis: *P < 0.05, **P < 0.01, ***P < 0.001. SEM, standard error of the mean. Figure S3. Positive scotopic threshold responses (pSTR). Bar charts comparing the amplitudes (a–d) and the implicit times (e–h) of the pSTR from db/ + mice (n = 14) and db/db mice (n = 11) at different experimental timepoints. Data presented as mean ± SEM. Simple main effect analysis: *P < 0.05, **P < 0.01, ***P < 0.001. SEM, standard error of the mean. [file 40662_2023_353_MOESM1_ESM.docx]
